# Analysis of pooled cross-sectional study data on smoking among pregnant and nursing mothers after a disaster: Pregnancy and birth survey of the Fukushima health management survey

**DOI:** 10.18332/tid/214490

**Published:** 2026-01-29

**Authors:** Hironori Nakano, Aya Goto, Kayoko Ishii, Miyuki Mori, Kohta Suzuki, Nihaal Rahman, Keiya Fujimori, Tetsuya Ohira, Seiji Yasumura

**Affiliations:** 1 Radiation Medical Science Center for the Fukushima Health Management Survey, Fukushima Medical University, Fukushima, Japan; 2 Department of Epidemiology, Fukushima Medical University School of Medicine, Fukushima, Japan; 3 Center for Integrated Sciences and Humanities, Fukushima Medical University, Fukushima, Japan; 4 Department of Global Health and Population, Harvard T.H. Chan School of Public Health, Boston, United States; 5 Department of Midwifery and Maternal Nursing, Fukushima Medical University School of Nursing, Fukushima, Japan; 6 Department of Maternal Nursing, Nagaoka Sutoku University School of Nursing, Niigata, Japan; 7 Department of Health and Psychosocial Medicine, Aichi Medical University School of Medicine, Aichi, Japan; 8 Department of Social and Behavioral Sciences, Harvard T.H. Chan School of Public Health, Boston, United States; 9 Department of Obstetrics and Gynecology, Fukushima Medical University School of Medicine, Fukushima, Japan

**Keywords:** pregnancy, smoking cessation, disasters

## Abstract

**INTRODUCTION:**

Japan is one of the countries most affected by both the global tobacco epidemic and disasters, which are often interrelated. This study aimed to analyze factors related to continuation of smoking or relapse after childbirth among women who smoked before pregnancy and inform approaches to help them continue smoking cessation in a post-disaster setting, such as that after the Fukushima nuclear accident.

**METHODS:**

We conducted a pooled analysis of secondary data collection from Fukushima prefecture-wide cross-sectional self-administered questionnaire-based surveys. Participants were recruited from women given a Maternal and Child Health Handbook by their city of residence in Fukushima Prefecture from 2013 to 2016. A total of 17211 responses to the Pregnancy and Birth Survey were analyzed. Women who smoked before pregnancy were divided according to smoking status during pregnancy and after childbirth, and then compared with those who did not smoke before pregnancy in terms of evacuation status, radiation risk perception, age, parity, subjective health, and depression tendency.

**RESULTS:**

A total of 16417 respondents did not smoke before pregnancy. Among those who smoked before pregnancy, 634 quit smoking during pregnancy and maintained cessation after childbirth, 182 quit smoking during pregnancy but relapsed afterward, 195 smoked during pregnancy but quit after childbirth, and 582 continued smoking during and after pregnancy. Age ≤24 years (AOR=2.36), multiparity (AOR=1.61), and depression tendency (AOR=1.85) were associated with relapse. Current evacuation status (AOR=1.65), radiation risk perception (AOR=0.55), age ≤24 years (AOR=2.19), multiparity (AOR=1.90), disease history (AOR=1.33), and depression tendency (AOR=1.85) were associated with continuation of smoking.

**CONCLUSIONS:**

Previous smokers who continue smoking or relapse after childbirth need support that addresses complex underlying factors, including mental health. Continuation of smoking was particularly associated with disaster-related factors, suggesting that disaster-affected mothers need multifaceted support for health promotion.

## INTRODUCTION

The Great East Japan Earthquake of 11 March 2011, and the accompanying Fukushima Daiichi nuclear accident severely affected the coastal region in Fukushima Prefecture and forced the long-term evacuation of many residents. Rain and wind carried the radiation plume from the nuclear power plant to the densely populated northern part of the prefecture^1^. In response, the prefecture government launched the Fukushima Health Management Survey (FHMS), a long-term epidemiological study investigating the health effects of prolonged exposure to low-dose radiation^2^.

Every year since 2011, Fukushima Medical University has conducted a Pregnancy and Birth Survey as part of the FHMS until 2024. This survey was conducted with new and expectant mothers who received a Maternal and Child Health Handbook from their municipal government. The aims of the survey were to assess the physical and mental health of mothers and children, reduce mothers’ anxiety around issues such as radiation exposure, and provide necessary care^3^. From 2011 to 2015, the survey was administered via mail and distribution at obstetric facilities; from 2016, an option to complete the survey online was added. Survey items covered maternal mental and physical health, child health, current living situation (e.g. evacuated or separated), obstetrical history, confidence in child-rearing, and intention to become pregnant again. Questions about smoking status were added in 2013^4^.

Japan remains one of the countries most affected by the global tobacco epidemic^5^. National data show a gradual decline in smoking among pregnant women and mothers of young children in Japan. According to the 2013 report for Healthy Japan 21 (2nd term), smoking rates were 3.8% among pregnant women and 8.1% among mothers of young children, compared with 2.7% and 6.4%, respectively, in the 2017 report^6^. Among the general population, the 2017 National Health and Nutrition Survey reported that 27.8% of men and 6.5% of women aged ≥20 years were current smokers^7^. While smoking rates among men have declined steadily, the rate among women has remained relatively unchanged. Evidence from the United Kingdom suggests that pregnancy may serve as a strong motivator for smoking cessation, as demonstrated by the 26% of former smokers who reduced their smoking frequency and the 35% who quit altogether during pregnancy^8^. However, the likelihood of quitting smoking due to pregnancy was lower among women who were younger, married due to pregnancy, came from low-income families with parents who smoked, and started smoking at a younger age. Similarly, a Japanese study found that 33.5% of women who quit smoking during pregnancy relapsed after giving birth, with mothers aged <28 years at the time of pregnancy registration being more likely to relapse^9^.

Smoking during pregnancy exposes the fetoplacental unit to nicotine and carbon monoxide, leading to hypoxia and increasing the risk of serious complications, including miscarriage, preterm delivery, low birth weight, impaired embryo-fetal development, and perinatal mortality. The effects extend to the newborn, including atopic dermatitis and respiratory illness^10-12^. Moreover, smoking has been shown to elevate concentrations of endotoxins linked to morning sickness by up to 30-fold^13^. Although reforms to Japan’s Health Promotion Act, implemented gradually from 2019, prohibit smoking in schools, hospitals, child welfare institutions, government offices, and large commercial establishments, these regulations do not address secondhand exposure in private residences, where pregnant women may remain vulnerable. In this context, obstetrician-gynecologists, working collaboratively with nurses and midwives, play a pivotal role in delivering continuous health promotion and preventive care to women and their families across the preconception, antenatal, and postpartum periods^14^. International guidelines, including those from the World Health Organization and the American College of Obstetrics and Gynecology, stress the importance of preconception care and periodic gynecological visits as opportunities for comprehensive behavioral risk assessment and intervention. Given the current limitations of legal protection against secondhand smoke in domestic environments in Japan, the clinical role of obstetrician-gynecologists is especially critical in advocating for and supporting tobacco-free homes during pregnancy.

While clinical support remains essential, it is also important to recognize that smoking behavior during pregnancy and postpartum is shaped by broader social and psychological stressors, including exposure to trauma and disaster-related mental health conditions. Post-disaster psychological distress can give rise to maladaptive coping behaviors, including problematic substance use such as smoking^15^. Various studies have examined the relationship between disaster-induced post-traumatic stress disorder (PTSD) and smoking behavior. In Japan, a study conducted using data from the Mental Health and Lifestyle Survey of the FHMS found that young age, low level of education, damage to one’s home, witnessing the tsunami, trauma symptoms, and nonspecific psychological distress, were associated with smoking initiation after the 2011 disaster^16^. Internationally, research on survivors of Hurricane Katrina demonstrated that increased rates of smoking relapse were mediated by heightened PTSD and depressive symptoms, and that social support played a protective role in reducing relapse likelihood^17^. Similarly, among survivors of the 9/11 World Trade Center attacks, those with higher PTSD were less likely to quit smoking, and although smoking rates decreased over time, they remained elevated among individuals with PTSD^18,19^. According to the national Comprehensive Survey of Living Conditions, the smoking rate among women in Fukushima Prefecture was 12.2% in fiscal year 2007, placing it 12th nationally, and decreased to 10.5% in fiscal year 2010, ranking 11th. However, following the earthquake, the smoking rate increased to 12.1% in 2013, moving up to 5th place, and subsequently fell to 10.1% in 2022, ranking 2nd nationwide^20^. This relative increase in the smoking rate suggests a potential impact of the 2011 earthquake.

Building on this evidence, we used FHMS data to examine factors associated with continued smoking and postpartum relapse among women who smoked before pregnancy, including exposure to disaster-related stressors. This analysis aims to inform approaches for supporting smoking cessation among new and expectant mothers in post-disaster settings.

## METHODS

### Study design and participants

We conducted a pooled analysis of secondary data collection from prefecture-wide cross-sectional self-administered questionnaire-based surveys. Participants for the 2013 to 2016 Pregnancy and Birth Surveys were recruited from: 1) women given a Maternal and Child Health Handbook by their city of residence in Fukushima Prefecture; and 2) women given a Maternal and Child Health Record Handbook by their city of residence outside of Fukushima Prefecture (i.e. women returning to their hometown to give birth) between 1 August of the survey year and 31 July of the following year in the 2013 to 2016 surveys.

The responses were analyzed after excluding non-responses, invalid, delayed, or duplicated responses, responses from women whose pregnancy did not result in a live birth or who returned to their hometown outside of Fukushima Prefecture to give birth, and responses filled in by someone other than the mothers.

**Figure 1:**
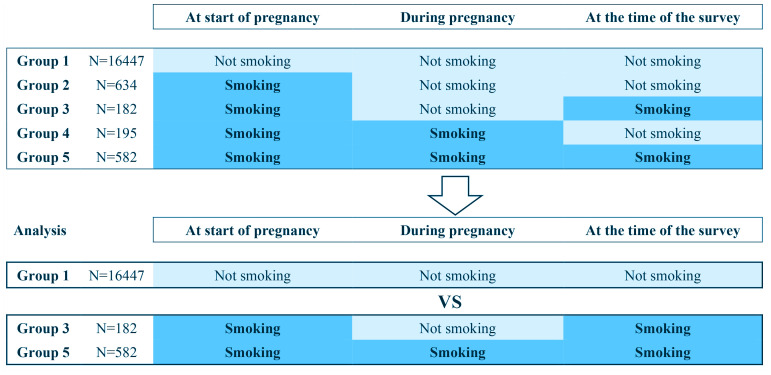
Grouping and analysis of participants

Respondents were divided into five groups as follows: 1) women who were non-smokers at the start of pregnancy and remained so during pregnancy and after childbirth; 2) women who were smokers at the start of pregnancy but quit during pregnancy and maintained cessation after childbirth; 3) women who were smokers at the start of pregnancy and quit during pregnancy but relapsed after childbirth; 4) women who were smokers at the start of pregnancy and during pregnancy but quit after childbirth; and 5) women who were smokers at the start of pregnancy, during pregnancy, and after childbirth (Figure 1).

### Survey methods

Women residing in Fukushima Prefecture received a notice regarding the FHMS Pregnancy and Birth Survey, a survey form, and instructions for responding online^21^ along with a leaflet about the previous survey results. Women returning to their hometown in Fukushima Prefecture to give birth were given a survey form when they visited their obstetric facility. Based on pregnancy registration information collected from municipalities, women were divided into three groups according to the expected date of delivery, so that the questionnaire would arrive at an appropriate time for them to answer it. Those who did not respond were sent a reminder postcard and a new survey form.

### Survey items

The survey items are as follows: 1) Currently living in an evacuation shelter at the time of the survey (designated and self-determined); 2) Concern about radiation risk (defined as bottle-feeding a baby and/or not intending to have the next pregnancy due to concerns about the effects of radiation); 3) Age (≤24, 25–29, 30–34, ≥35 years); 4) Parity (primipara, multipara); 5) Disease history (suffer from any disease prior to the current pregnancy); and 6) Depressive tendencies. Depressive tendencies were assessed using a two-item screening tool developed by Mishina et al.22 to identify postpartum depression. In this survey, participants were asked: ‘During the past month, have you often felt down, depressed, or hopeless?’ and ‘During the past month, have you often found little interest or pleasure in doing things?’. Mothers who answered ‘yes’ to one or both questions were classified as positive for depressive symptoms. It was reported that using the Edinburgh Postpartum Depression Scale (EPDS) as the standard, the sensitivity and specificity of the two-item screening tool were 88% and 76%, respectively^22^.

### Analysis

Only data with responses to all items required for analysis were analyzed. First, we reported basic descriptive statistics using the proportions of various characteristics among Groups 1, 3, and 5. Next, bivariable logistic regression analysis was used to calculate odds ratios by using the survey items listed above. The survey year was added, as this was an analysis of pooled data. Finally, we used multivariable logistic regression analysis to investigate associations between non-smoking and relapse or continuation of smoking for significant factors in the bivariable analysis. We compared Group 1 with Groups 3 and 5 to identify the factors associated with relapse and continuation of smoking. Specifically, we conducted analyses examining factors related to the 2011 disaster (evacuation status, radiation risk perception) and factors previously associated with smoking [age, parity, and mental health (depressive tendencies)]^9,11,15-18^. A p<0.05 was considered statistically significant. The analysis was performed using a two-tailed test. SAS version 9.4 (SAS Institute, Cary, NC) was used for all analyses.

### Ethical considerations

This study was conducted with the approval of the institutional review board of Fukushima Medical University (Approval numbers: 1317 & 2333; Dates: 15 September 2011 & 21 December 2017). The purpose of the survey was explained on a written information sheet attached to the survey form, and responding to the survey was considered to indicate consent to participate. Written information was also displayed on the online survey webpage, and clicking the ‘send response’ button was considered to indicate consent to participate.

## RESULTS

The total number of responses to the survey in 2013, 2014, 2015, and 2016 was 7122, 7023, 6914, and 7184, respectively. A total of 17211 responses were analyzed. The number of women who did not smoke before pregnancy (Group 1) was 16447. Among those who smoked before pregnancy, 634 quit smoking during pregnancy and maintained cessation after childbirth (Group 2), 182 quit smoking during pregnancy but relapsed afterward (Group 3), 195 smoked during pregnancy but quit after childbirth (Group 4), and 582 continued smoking during and after pregnancy (Group 5) (Figure 1).

Table 1 displays the proportions of various characteristics across the groups. The proportion still living in an evacuation shelter was lower in Group 1 (4.1%) compared to Group 3 (6.6%) and Group 5 (7.0%). Radiation risk perception was higher in Group 1 (20.6%) but lower in Group 3 (16.5%) and Group 5 (11.2%). The proportion of individuals aged ≤24 years was lower in Group 1 (13.8%), whereas Group 3 (23.1%) and Group 5 (22.9%) had higher proportions. The proportion of primiparas was higher in Group 1 (51.9%) than in Groups 3 (42.3%) and 5 (36.6%). The proportion with a disease history was higher in Groups 3 (34.1%) and 5 (34.9%) compared to Group 1 (28.9%). Finally, the proportion with depressive tendencies was lower in Group 1 (21.0%) but higher in Groups 3 (33.0%) and 5 (33.3%).

**Table 1:** Characteristics of participants

*Characteristics*	*Group 1 (N=16447)*		*Group 3 (N=182)*		*Group 5 (N=582)*	
	*Not smoking at start of pregnancy*		*Not smoking during pregnancy but smoking after childbirth (relapse)*		*Smoking before pregnancy, during pregnancy, and after childbirth (continuation)*	
	*n*	*%*	*n*	*%*	*n*	*%*
**Currently living in an evacuation shelter**						
Yes	682	4.1	12	6.6	41	7.0
No	15765	95.9	170	93.4	541	93.0
**Concern about radiation risk**						
Yes	3385	20.6	30	16.5	65	11.2
No	13062	79.4	152	83.5	517	88.8
**Age** (years)						
≤24	2263	13.8	42	23.1	133	22.9
25–29	4348	26.4	45	24.7	126	21.6
30–34	5560	33.8	46	25.3	165	28.4
≥35	4276	26.0	49	26.9	158	27.1
**Parity**						
Primipara	8533	51.9	77	42.3	213	36.6
Multipara	7914	48.1	105	57.7	369	63.4
**Disease history**						
Yes	4747	28.9	62	34.1	203	34.9
No	11700	71.1	120	65.9	379	65.1
**Depression tendency**						
Yes	3447	21.0	60	33.0	194	33.3
No	13000	79.0	122	67.0	388	66.7

Table 2 presents the factors associated with relapse and continuation of smoking, based on bivariable analysis. When comparing Group 1 with Group 3, after adjusting for survey year, factors such as age ≤24 years (adjusted odds ratio, AOR=2.24; 95% CI: 1.47–3.42, ref. 30–34 years), multiparity (AOR=1.47; 95% CI: 1.09–1.98, ref. primiparity), and depressive tendencies (AOR=1.86; 95% CI: 1.36–2.53, ref. no) were associated with smoking relapse. In the comparison between Group 1 and Group 5, current evacuation status (AOR=1.75; 95% CI: 1.26–2.43, ref. no), radiation risk perception (AOR=0.49; 95% CI: 0.37–0.63, ref. no), age ≤24 years (AOR=1.98; 95% CI: 1.57–2.50, ref. 30–34 years), multiparity (AOR=1.87; 95% CI: 1.57–2.22, ref. primiparity), disease history (AOR=1.32; 95% CI: 1.11–1.57, ref. no), and depressive tendencies (AOR=1.89; 95% CI: 1.58–2.25, ref. no) were associated with the continuation of smoking.

**Table 2:** Bivariate analysis of factors associated with relapse and continuation of smoking

*Variables*	*Group 1 vs Group 3*			*Group 1 vs Group 5*		
	*(Non-smoking vs relapse)*			*(Non-smoking vs continuation)*		
	*AOR*	*95% CI*	p*	*AOR*	*95% CI*	p*
**Currently living in an evacuation shelter**						
No ®	1.00			1.00		
Yes	1.63	0.90–2.95	0.104	1.75	1.26–2.43	<0.001
**Concern about radiation risk**						
No ®	1.00			1.00		
Yes	0.76	0.51–1.13	0.175	0.49	0.37–0.63	**<0.001**
**Age** (years)						
30–34 ®	1.00			1.00		
≤24	2.24	1.47–3.42	**<0.001**	1.98	1.57–2.50	**<0.001**
25–29	1.25	0.83–1.89	0.288	0.98	0.77–1.24	0.843
≥35	1.39	0.92–2.08	0.114	1.25	1.00–1.55	0.053
**Parity**						
Multipara	1.47	1.09–1.98	**0.011**	1.87	1.57–2.22	**<0.001**
Primipara ®	1.00			1.00		
**Disease history**						
Yes	1.27	0.94–1.73	0.124	1.32	1.11–1.57	**0.002**
No ®	1.00			1.00		
**Depressive tendency**						
No ®	1.00			1.00		
Yes	1.86	1.36–2.53	**<0.001**	1.89	1.58–2.25	**<0.001**

AOR: adjusted odds ratio, adjusted by survey year.

* Numbers in bold indicate statistical significance at p<0.05. ® Reference categories.

Table 3 presents the multivariable analyses. When comparing Group 1 with Group 3, the analysis revealed that age ≤24 years (AOR=2.36; 95% CI: 1.54–3.61, ref. 30–34 years), multiparity (AOR=1.61; 95% CI: 1.19–2.18, ref. primiparity), and depressive tendencies (AOR=1.85; 95% CI: 1.35–2.53, ref. no) were associated with smoking relapse. In the comparison between Group 1 and Group 5, current evacuation status (AOR=1.65; 95% CI: 1.19–2.29, ref. no), radiation risk perception (AOR=0.55; 95% CI: 0.420.72, ref. no), ≤24 years (AOR=2.19; 95% CI: 1.73–2.78, ref. 30–34 years), multiparity (AOR=1.90; 95% CI: 1.59–2.27, ref. primiparity), disease history (AOR=1.33; 95% CI: 1.12–1.59, ref. no), and depression tendency (AOR=1.85; 95% CI: 1.54–2.21, ref. no) were associated with the continuation of smoking.

**Table 3:** Multivariable analysis of factors associated with relapse and continuation of smoking

*Variables*	*Group 1 vs Group 3 (Non-smoking vs relapse)*			*Group 1 vs Group 5 (Non-smoking vs continuation)*		
	*AOR*	*95% CI*	p*	*AOR*	*95% CI*	p*
**Currently living in an evacuation shelter**						
No ®				1.00		
Yes				1.65	1.19–2.29	**0.003**
**Concern about radiation risk**						
No ®				1.00		
Yes				0.55	0.42–0.72	**<0.001**
**Age** (years)						
30–34 ®	1.00			1.00		
≤24	2.36	1.54–3.61	**<0.001**	2.19	1.73–2.78	**<0.001**
25–29	1.34	0.88–2.02	0.173	1.11	0.87–1.40	0.406
≥35	1.34	0.89–2.01	0.159	1.14	0.91–1.42	0.259
**Parity**						
Multipara	1.61	1.19–2.18	**0.002**	1.90	1.59–2.27	**<0.001**
Primipara ®	1.00			1.00		
**Disease history**						
Yes				1.33	1.12–1.59	**0.001**
No ®				1.00		
**Depressive tendency**						
No ®	1.00			1.00		
Yes	1.85	1.35–2.53	**<0.001**	1.85	1.54–2.21	**<0.001**

AOR: adjusted odds ratio, adjusted for items listed above and survey year.

* Numbers in bold indicate statistical significance at p<0.05. ® Reference categories.

## DISCUSSION

In the present study, younger age, parity, and the mental health of mothers were associated with either or both of smoking continuation and relapse. Other studies in Japan have reported that multiparity, younger age, and depression tendency lasting 2 weeks or longer might be risk factors for smoking relapse after childbirth^8,12,23^. Reviews of both quantitative and qualitative studies outside Japan have identified maternal age, age at the start of smoking, severity of addiction, partner’s smoking, secondhand smoking, socioeconomic status, education level, parity, mental health, and baby feeding method as factors associated with continuation of smoking after childbirth^24-26^. Our findings are in line with those of these previous studies.

When we compared women who were non-smokers at the start of pregnancy with women who continued smoking during pregnancy and after childbirth, we found that low radiation risk perception was associated with continued smoking. Such low risk perception toward an environmental risk may indicate mothers’ underlying disregard of their own health as well as that of their children. This might be reflected in the disease history being associated with continued smoking. Among the general public in Fukushima, Kashiwazaki et al.^27^ reported that radiation anxiety and health anxiety are associated, and they too suggested the need for interventions to alleviate health anxiety in general rather than focusing solely on radiation anxiety. With regard to smoking during pregnancy, studies from Europe and North America have shown its association with drinking during pregnancy, suggesting that these women have low perception of health risks overall^28,29^. Taken together with the fact that smoking during pregnancy is associated with low maternal health literacy^30^, health professionals should address overall health promotion of women in the perinatal period.

Another factor related to the 2011 disaster associated with continued smoking was evacuation status, which was suggested by our group’s previous study focusing on smoking relapse and midwifery^23^. As seen in past major earthquakes, many residents of areas affected by the Great East Japan Earthquake experienced stress-induced hypertension and persistent worsening of chronic diseases^31^. On a positive note, the FHMS Mental Health and Lifestyle Survey showed that the number of women from evacuation zones who smoked decreased from 10.5% in 2011 to 6.8% in 2017^32^. Nevertheless, psychological stress caused by a disaster may lead to the development of diseases both directly and indirectly through negative health behavior changes^15^.

### Limitations

Our study had several methodological limitations. First, this study analyzed pooled data from cross-sectional surveys, and we could not establish causal relationships. Second, this study uses self-administered questionnaires, which may introduce information biases such as recall bias, social desirability bias, and misclassification. Third, because our survey used a self-completed form, health-conscious women might be more likely to respond, which could result in a lower response rate among smokers. However, this would not impact the internal validity of the results on factors related to smoking. Fourth, despite identifying changes in smoking status during pregnancy and after childbirth, we did not examine changes in smoking frequency. Fifth, this study suffers from residual confounding. More specifically, information on several factors reported to be associated with mothers’ smoking was missing in our database, the primary goal of which was to alleviate mothers’ anxiety after the nuclear accident. These included the smoking status of the father and the maternal grandparents, as well as socioeconomic status. Previous studies from Japan, for instance, have identified low economic status, low household income, and partner smoking status as significant factors associated with maternal smoking^33^. Given that smoking cessation by one’s partner may prevent maternal smoking^34^, educational efforts must also target family members. In addition, only about 12% of medical institutions in Japan offer smoking cessation counseling or treatment programs, highlighting the need for broader implementation of these services nationwide^14^. Epidemiologically, it is clear that more rigorous research is needed to understand the complex factors underpinning mothers’ continuation and relapse of smoking. Furthermore, the survey form did not define e-cigarettes and only asked about classical smoking, specifically whether respondents smoked tobacco. Finally, this study focuses on a specific group of respondents under the unique circumstances of Japan’s combined disaster involving earthquake, tsunami, and radiation, and therefore cannot be generalized. Despite these limitations, our findings offer insights into the factors influencing smoking behavior in the context of disaster-related stress and maternal health, providing a foundation for future research in this area.

## CONCLUSIONS

We found that younger maternal age, multiparity, depressive tendencies, radiation risk perception, and ongoing disaster-related evacuation were significantly associated with smoking relapse and continuation during the perinatal period. Further longitudinal studies in disaster-affected regions of Japan are warranted to clarify causal pathways and inform targeted, community-based interventions. In light of the increasing number of climate change-related disasters and humanitarian crises worldwide, these findings may also have broader implications for health promotion among displaced populations.

## Data Availability

The data supporting this research are available from the authors on reasonable request.
